# Cortical Transformation of Spatial Processing for Solving the Cocktail Party Problem: A Computational Model^1^,^2^,^3^

**DOI:** 10.1523/ENEURO.0086-15.2015

**Published:** 2016-02-02

**Authors:** Junzi Dong, H. Steven Colburn, Kamal Sen

**Affiliations:** Hearing Research Center and Department of Biomedical Engineering, Boston University, Boston, Massachusetts 02215

**Keywords:** auditory model of spatial processing, cocktail party problem, computational modeling, spatial auditory processing, spatial segregation

## Abstract

In multisource, “cocktail party” sound environments, human and animal auditory systems can use spatial cues to effectively separate and follow one source of sound over competing sources. While mechanisms to extract spatial cues such as interaural time differences (ITDs) are well understood in precortical areas, how such information is reused and transformed in higher cortical regions to represent segregated sound sources is not clear. We present a computational model describing a hypothesized neural network that spans spatial cue detection areas and the cortex. This network is based on recent physiological findings that cortical neurons selectively encode target stimuli in the presence of competing maskers based on source locations (Maddox et al., 2012). We demonstrate that key features of cortical responses can be generated by the model network, which exploits spatial interactions between inputs via lateral inhibition, enabling the spatial separation of target and interfering sources while allowing monitoring of a broader acoustic space when there is no competition. We present the model network along with testable experimental paradigms as a starting point for understanding the transformation and organization of spatial information from midbrain to cortex. This network is then extended to suggest engineering solutions that may be useful for hearing-assistive devices in solving the cocktail party problem.

## Abstract (precis)

In multisource, “cocktail party” sound environments, human and animal auditory systems can use spatial cues to effectively separate and follow one source of sound over competing sources.

## Visual Abstract

**Figure:**
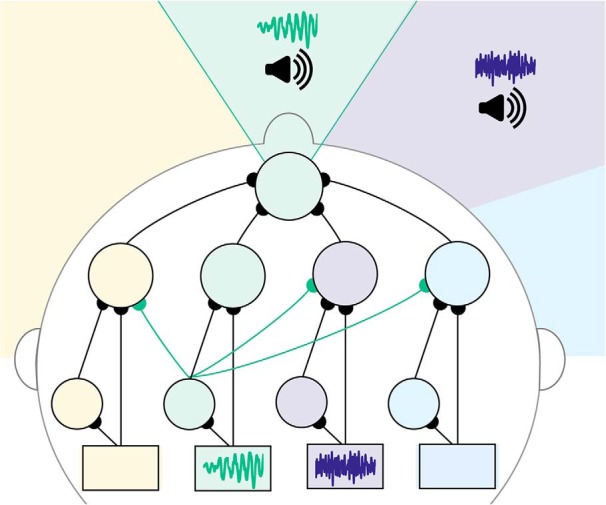

## Significance Statement

Spatial cues are known to be critical for human and animal brains when following specific sound sources in the presence of competing sounds, but the exact mechanism by which this happens is not clear. The role of spatial cues in localizing single sound sources in the midbrain is well documented, but how these extracted cues are used downstream in the cortex to separate competing sources is not clear. We present a computational neural network model based on recent recordings to bridge this gap. The model identifies specific candidate physiological mechanisms underlying this process and can be extended to construct engineering solutions that may be useful for hearing assistive devices for coping with the cocktail party problem.

## Introduction

The problem of recognizing and processing individual auditory objects in complex listening environments, the “cocktail party problem”, was recognized over 50 years ago (Cherry, 1953); however, its neural mechanism remains poorly understood. Human and animal auditory systems selectively segregate and follow a selected sound source in the presence of competition to make sense of multiple-source environments (Bregman, 1994). Spatial cues enable listeners to segregate and follow individual sources, as demonstrated by human and animal studies (Hine et al., 1994; Dent et al., 1997, 2009; Darwin and Hukin, 1998; Arbogast et al., 2002). Although precortical neurons have been extensively shown to be selectively tuned to spatial cues, such as interaural time difference (ITD; Knudsen and Konishi, 1978; Yin and Chan, 1990; Peña and Konishi, 2001; Köppl and Carr, 2008; Devore et al., 2009), how spatial information from spatial cue detection areas is relayed to and used in higher cortical areas is not clear (Vonderschen and Wagner, 2014). Recent experiments on cortical responses revealed that whereas spatial tuning for single sound sources is broad, simultaneous competing sources increase spatial selectivity (Maddox et al., 2012; Middlebrooks and Bremen, 2013). Although these findings shed light on the spatial encoding capabilities of the cortex, neural mechanisms capable of generating such capabilities remain unknown. The goal of this study is to provide a computational model consistent with existing physiological evidence to describe the transformation between precortical areas and the cortex, which can selectively encode target stimuli when presented with competing sources in space. Specifically, we present a model network that replicates the spatial responses observed in a study by Maddox et al. (2012), providing a mechanistic solution to the spatial segregation of independent sources.

**Figure 1.**
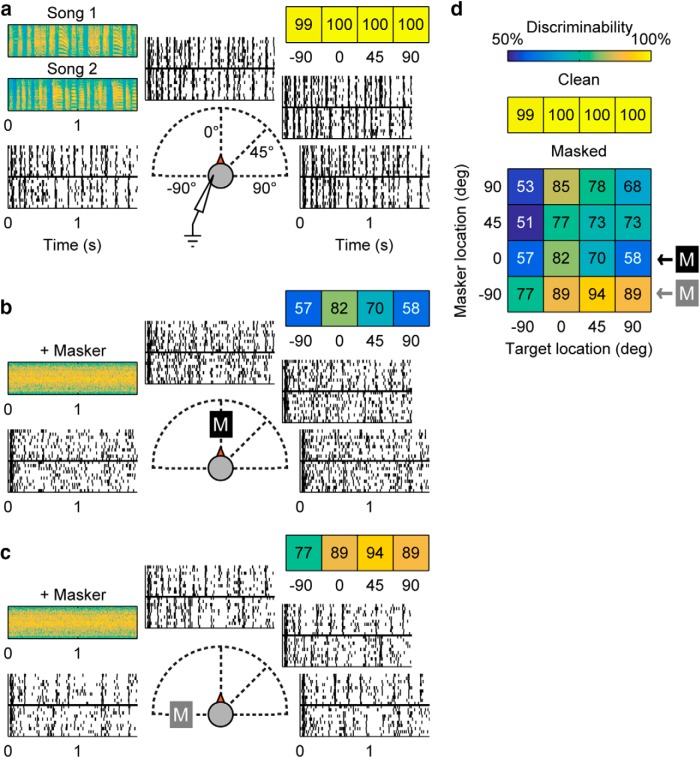
Recorded cortical neurons develop sharper spatial responses to targets when a noise masker is present (Maddox et al., 2012). ***a***, Responses to target alone. Two bird songs—Song 1 and 2 (spectrograms shown in top left)—are played separately from four locations −90°, 0°, 45°, and 90°. Recorded raster plots of responses to the two birdsongs are shown at each azimuth location. Positive degrees indicate locations contralateral to recording site. The color-coded discriminability values for each location are shown in the horizontal grid on the upper right. (Color map for all panels is shown in ***d***, top row.) ***b*, *c***, Responses to target with masker. Masker and one target song are played concurrently from one (colocated) or two (separated) of the four stimulus locations. A masker fixed at 0° or −90°, indicated by a black or grey boxed M, respectively, whereas the target song is played at one of the locations shown. As in ***a***, recorded raster responses from each target location are shown, and discriminability values are shown in the colored grid of values (top right). ***d***, Discriminability values for all location combinations. The top grid (single row) of numbers are the discriminability values for the “clean” (target-alone) conditions. In the lower, spatial discriminability grid, each block indicates a target and masker location combination. The rows indicated by a black or grey boxed M are cases where the masker is fixed at 0° or −90°. Blocks in all grids are colored according to the color scale given at the top of this panel.

Maddox et al. (2012) demonstrated that, although the coding of song identity is not strongly impacted by stimulus location in quiet, location does have a significant effect on neural coding when there is a competing masker. In their experiments, two birdsongs were first presented independently from one of four stimulus locations (Fig. 1*a*). The neuron’s spatial performance was studied using the discriminability index, a metric quantifying the neural coding of song identity at each location. A larger difference in neural responses to the two songs gives higher song discriminability, indicating a location where birdsong is more “intelligible” to the neuron. For the target song alone (“clean”) case, similar discriminability across locations (Fig. 1*a*) indicates broad spatial tuning, where all spatial locations are similarly encoded within this neuron. In the masked conditions illustrated in Figure 1, *b* and *c*, a noise masker is played concurrently with a target, and the two are covaried in location for all possible combinations. A spatial discriminability grid of responses to all recorded target and masker location combinations (Fig. 1*d*) shows that for this unit, discriminability is better at a few “hotspots” shaded in lighter colors. These patterns indicate a sharpened spatial preference for encoded song stimuli in the presence of a competing masker at these locations.

In this paper, our goals are to construct a model network capable of replicating key features of the experimentally observed cortical responses: (1) similar discriminability for target songs in quiet at any location, indicating broad tuning and the ability of neurons to monitor the entire acoustic space in quiet; and (2) the emergence of hotspots where coding of song identity is enhanced at select stimulus locations in the presence of a second competing sound (the masker). The network can be adjusted to model a diverse range of spatial responses, demonstrated by fitting the population of neurons reported in the Maddox et al. (2012) study. Finally, we propose a way to extend this network to design engineering solutions that may be useful for achieving spatial stream segregation in hearing-assistive devices.

## Methods

### Network model overview

The network is composed of a three-layer structure, where the bottom layer receives precortical input, and the final layer provides the cortical output, which is then compared to the recordings. The model architecture, model mechanisms and parameters, and simulated precortical input are explained in separate sections below.

### Network model architecture

**Figure 2.**
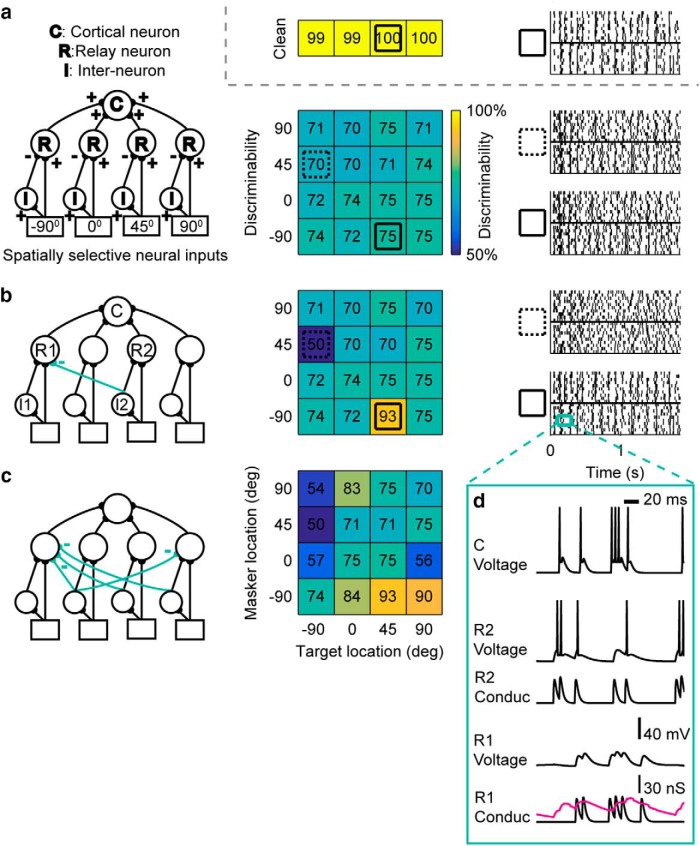
Lateral inhibition in the model can account for the spatial tuning and spatial segregation properties of recorded units. ***a***–***c***, Left, Model structure. Center, Simulated spatial grid. Right, Raster plots for stimulus conditions indicated by dashed or solid squares in the grid. Top right, Inset, The simulated discriminability for the clean (no-masker) case indicating broad spatial tuning. This clean case is not impacted by the addition of lateral inhibition, and is identical for all networks shown. ***a***, Basic model structure with no lateral inhibitory connections. Simulated multisource spatial grid in model without lateral inhibition lacks the spatial diversity observed in the data. ***b***, Spatial grid produced by the model with one inhibitory connection between 0° and −90°, shows an increase in discriminability when target and masker are presented at 0° and −90°, respectively. ***c***, Model with additional inhibitory connections simulates the spatial response of the recorded unit shown in Figure 1*d*. ***d***, Subthreshold responses of relay and cortical neurons, R1, R2, and C (***b***, left), for the labeled time segment (***b***, right) of one trial when target is presented at 0° and masker at −90°. Direct excitatory currents to R1 (R1 Conduc: black curve) are offset by inhibitory currents from I2 (R1 Conduc: magenta curve), and R1 is unable to reach spiking threshold, as seen in its voltage trace (R1 Voltage: black curve). In contrast, R2 is able to relay its temporal information to C, whose spiking pattern (C Voltage) resembles that of R2 (R2 Voltage).

The structure of the model, which was custom written in MATLAB, can be seen in Figure 2*a*–*c*. The basic architecture consists of an input layer with four spatial input channels corresponding to −90°, 0°, 45°, and 90° to mirror the experimental design of Maddox et al. (2012), and an intermediate layer of processing that includes excitatory relay neurons (R) and inhibitory neurons (I), and an output cortical neuron (C). The detailed network connectivity is determined by the additional lateral inhibitory connections as illustrated in Figure 2. Our goal was to match the response of the output cortical neuron C in the model to the main features of the neurons recorded in the experiments by Maddox et al. (2012).

#### Biological rationale

The convergence architecture was hypothesized based on physiological data showing selected spatial tuning responses in the midbrain (Knudsen and Konishi, 1978; Yin and Chan, 1990; Köppl and Carr, 2008), in contrast to the broad tuning observed in the cortex (Stecker et al., 2005; Higgins et al., 2010). The spectrotemporal response properties of the input layer neurons were modeled after experimentally measured spectrotemporal receptive fields (STRFs) of neurons in the avian midbrain (Amin et al., 2010; see Network model input). We modeled four spatial input channels as described above. In the biological system, there could be more input channels tuned at different locations at a finer spatial resolution. The spatial tuning of zebra finch midbrain neurons remains unknown. We began with the simplest assumption that there were no interactions across spatial input channels, and later relaxed this assumption to allow spatial overlap between the input channels and demonstrated that the model remains robust over a range of spatial overlaps (see Spatial tuning width at the input stage and Fig. 4).

This model architecture is consistent with the inhibitory (and relay) neurons being located anywhere in the processing stream between the input (midbrain) neurons and the output cortical neuron. It is possible that the inhibitory (and relay) neurons are located in the thalamus. Inhibitory neurons have been found at the thalamic level in birds (Pinaud and Mello, 2007) and some mammals (Winer, 1992). Alternatively, inhibitory (and relay) neurons might be located within cortex prior to the output cortical neuron. There is extensive evidence supporting the presence of inhibitory neurons at the cortical level, both in birds and mammals (Pinaud and Mello, 2007; Oswald et al., 2006).

### Model neurons

All neurons in the model are integrate-and-fire neurons. Specific parameters used are described below. Resting potential was −60 mV, spiking threshold was −40 mV, and the reversal potential for excitatory currents was 0 mV for all neurons. In relay neurons, the reversal potential for inhibitory currents was −70 mV. In interneurons, EPSC was modeled as an alpha function with a time constant of 1 ms. In relay neurons, both EPSC and IPSC were modeled as the difference of a rising and a falling exponential, where rise and fall time constants were 1 and 3 ms, and 4 and 50 ms, respectively. An absolute refractory period of 3 ms was enforced in all neurons. These values are physiologically plausible (Froemke et al., 2007). In the cortical neuron, spike-rate adaptation was implemented by a hyperpolarizing conductance term that increases after firing and then recovers to zero exponentially (Dayan and Abbott, 2001). The adaptation time constant was 400 ms, and the strengths of the adaptation conductance for simulated neural units are shown in Table 1. Input synapses to the cortical neuron also have synaptic depression, which were modeled as described by Varela et al. (1997). Although this quantitative formulation was applied to visual cortical synapses in Varela et al. (1997), synaptic depression is also observed in auditory thalamocortical circuits (Atzori et al., 2001; Rose and Metherate, 2005; Oswald et al., 2006; Levy and Reyes, 2012). We used a single synaptic depression component with fixed time course of 80 ms, and synaptic depression factor of 0.95, to model the experimental data by Maddox et al. (2012). Both adaptation and synaptic depression were implemented in the simulations shown in Figures 2 and 4 for all modeled neurons.

**Table 1.** STRFs input and adaptation conductance used for each simulated neural unit

STRF no.	Neural units	Adaptation conductance
1	3, 6, 9, 10, 11, 13, 21, 23	0.025
	14, 22	0.04
2	15	0
3	29	0.12
	27	0.1
4	7	0.07
5	19	0.06
6	2	0.06
7	5	0.2
	25	0.16
8	20, 32	0.07
	1, 12, 33	0.08
9	16, 23	0.09
10	8	0.09
11	4	0.09
12	26, 28, 31	0.03
13	17	0.01
	18	0.03

STRF input and adaptation conductance were fit to best match the firing characteristics of each neuron recorded in the Maddox et al. (2012) study, whereas other neuron modeling parameters were fixed as reported above.

### Parameter fitting

Parameters were held constant throughout all simulations, except for the synaptic strengths and the strength of neural adaptation. To fit each recorded neuron, we first fit the general neural dynamics and baseline discriminability values by adjusting the strength of neural adaptation and the synaptic strengths without lateral inhibition. The specific values of neural adaptation used can be found in Table 1. The feedforward synaptic weights (input to relay neuron) were then adjusted to match the discriminability values for clean and co-located cases at each azimuth, whereas other parameters were held the same. For lateral inhibition, the synaptic strength of each inhibitory connection was chosen to model the recorded discriminability of its corresponding song and masker location. Our goal in this study was to fit the spatial discriminability grids observed experimentally.

### Network model input

**Figure 3.**
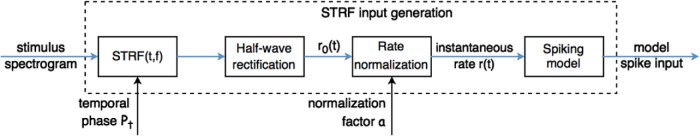
Illustration of model input generation process. The stimulus spectrogram was convolved with STRFs modeled after midbrain neurons, followed by half-wave rectification, then rate normalization to generate an instantaneous output-firing rate. This firing rate was then used to generate spikes using a spiking model (see Materials and Methods for details). The values of temporal phase *P_t_* and normalization factor a used were reported in Table 2.

**Figure 4.**
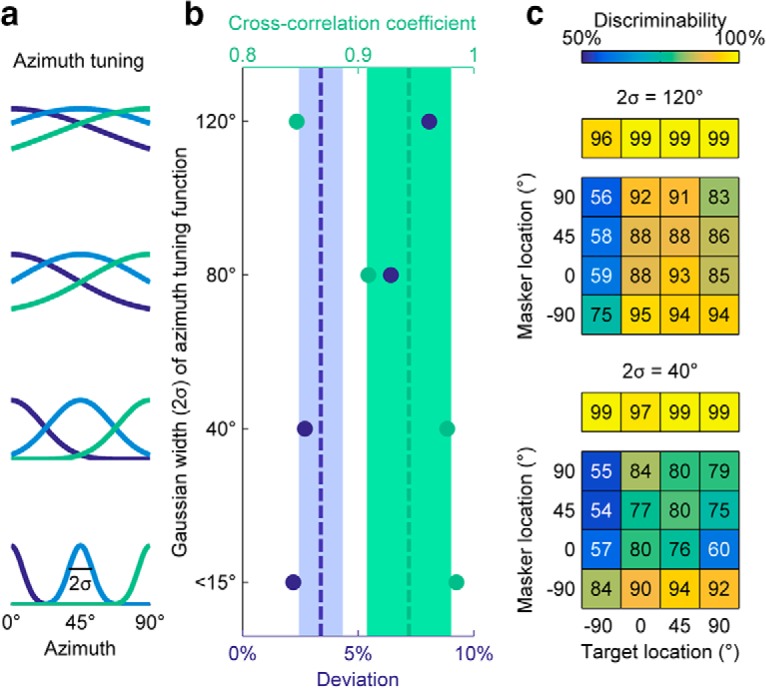
Network performance is robust to broader spatial tuning of inputs, as shown by extended simulations on the example unit previously displayed in Figure 2. ***a***, Illustrations of Gaussian spatial tuning curves of varying widths, defined by twice the standard deviation (2σ). ***b***, Results of spatial grid simulations for broadened input tuning width 2σ at 40°, 80°, and 120°, compared with the no-overlap case (<15°) on the bottom. The cross-correlation coefficient and deviation of the simulated results are plotted in green and purple, respectively, on separate horizontal axes. On the cross-correlation coefficient axis (top), larger values (closer to unity) indicate a better fit, whereas the deviation axis (bottom) shows better fits at smaller values closer to 0%. For reference, shaded areas and dotted lines indicate the mean and standard deviation of cross-correlation coefficient and deviation values, for original simulated population using non-overlapping inputs. As the spatial tuning of input units was broadened from <15° to 120°, the correlation coefficient (green dots) and the deviation (purple dots) degraded gracefully. The correlation coefficient remained above 0.8 and the deviation remained below 10% for the broadest tuning width. ***c***, Illustrations of simulated spatial grids with input widths of 40° and 120°. The 40° spatial grid can be compared with the no overlap spatial grid shown in Figure 2*c*. The two grids show a similar visual pattern, which is quantified by the similar deviation and cross-correlation coefficient values shown in ***b***. The 120° grid maintains the general pattern but has overall higher discriminability throughout.

The model input is composed of four spatial input channels corresponding to the stimulus locations used in the experiment by Maddox et al. (2012) (Fig. 2). Each channel receives simulated spike train responses of neurons at midbrain level as input. Input responses were simulated with STRFs modeled after typical STRFs obtained from the midbrain (MLd) of zebra finch songbirds (Amin et al., 2010). The input generation process is illustrated in Figure 3 and explained in detail below. For the majority of simulations, the azimuth response field for each modeled neuron was simulated with a Gaussian function, and across the population, there was minimal overlap between response fields. (Fig. 4, bottom left). This no-overlap assumption effectively means that for the azimuth locations used in the experiment, neighboring sources are outside the spatial receptive field, and each input channel will only respond to stimuli from its corresponding location. The effect of wider spatial tuning was also studied by running separate simulations with wider, overlapping Gaussian inputs (see Results).

#### Model input using STRFs

STRFs were used to simulate input responses. These STRFs were modeled using the product of Gabor functions in the time and frequency domain (Qiu et al., 2003):

STRF(t,f)=G(f) ⋅ H(t), where

$$G(f)=e^{-0.5[(f-f_{0})/\sigma_{f}]}{}^{{}^{2}}\;\cdot \;\cos[2\pi \;\cdot \;\Omega_{f}\;\cdot \;(f-f_{0})],\;and$$

$$H(t)=e^{-0.5[(t-t_{0})/\sigma_{t}]}{}^{{}^{2}}\;\cdot \;\cos[2\pi \;\cdot {\;\Omega }_{t}\;\cdot \;(t-t_{0})+P_{t}].$$

The frequency range is determined by $f_{0}$, the best frequency; $\sigma_{f}$, the spectral bandwidth; and $\Omega_{f}$, the best spectral modulation frequency, which were chosen and fixed at 4300 Hz, 2000 Hz, and 50 μs, respectively, to generate a broadband STRF for all simulations based on physiological ranges reported in the MLd of zebra finch songbirds by Amin et al. (2010). Temporal parameters $t_{0}$, the temporal latency; $\sigma_{t}$, the temporal bandwidth; and $\Omega_{t}$, the best temporal modulation frequency, were assigned 7 ms, 4.5 ms, and 56 Hz, respectively, based on recorded physiological values (Amin et al., 2010).

The normalization factor and temporal phase ($P_{t}$) were varied to match the neuron-specific raster responses seen in the neural recordings of the Maddox et al. (2012) study. Other STRF parameters were largely fixed for simplicity, but the model is robust to variations in these parameters. Specific values of used parameters are shown in Table 2.

**Table 2.** Parameters used for each type of input model STRF

STRF no.	Normalization factor	$P_{t}$ (rad)
1	0.08	1.4608
2	0.1	1.4923
3	0.07	1.508
4	0.1	
5	0.12	1.5237
6	0.1	1.5394
7	0.07	1.5425
8	0.087	
9	0.15	1.5582
10	0.05	
11	0.08	
12	0.16	1.5598
13	0.17	1.5708

Temporal phase $P_{t}$ and normalization factor are adjusted to match the recorded responses of the corresponding neurons, while other temporal and spectral parameters are held fixed and reported above.

#### STRF modeled input spike trains

As shown in Figure 3, STRFs were first converted to firing rates by convolving the stimulus spectrogram with the model STRF and half-wave rectifying so that rate outputs were positive. For each simulated neuron, the firing rate was normalized by factor a to adjust the final mean firing rate: $r(t)=a\cdot r_{0}(t)$. Finally, a Poisson spike model with a refractory period of 6 ms generated the neural response spikes used as the network model inputs, consistent with the instantaneous rates.

#### Spatial tuning width at the input stage

Spatial tuning width at the midbrain level varies across species, and is notably broader relative to the behavioral tuning for some mammals (Vonderschen and Wagner, 2014). To investigate whether the network model is functionally feasible with broader spatial tuning, the effect of spatial tuning width variation was studied by running simulations on an example neural unit and its neural network. The spatial tuning curves of input neurons were assumed to be Gaussian functions with varying standard deviations (SD), as shown in Figure 4. Tuning widths (twice the SD σ) of 15° or smaller result in no crosstalk between the input channels separated by 45°, as implemented in the main experiment. For the model unit used to test the effect of overlap (Table 1, unit 2), the tuning was then increased to show differences in model responses.

### Discriminability index: evaluating stimulus encoding and spatial tuning

The discriminability index calculates the level of dissimilarity between spike trains generated in response to two songs (Wang et al., 2007). For both sets of ten spike trains recorded from the same neuron, a random spike train from each song is chosen as a template, and the remaining spike trains are assigned to the closest template based on the van Rossum spike distance metric, which measures discrimination between two spike trains (van Rossum, 2001). This yields a perfect discriminability of 100% for an ideal response pair, and a chance discriminability of 50% for an indiscriminable response pair.

## Quantifying goodness of fit

To assess the fit of the model to individual units from the original study, we calculated the average deviation and correlation coefficient between the discriminability values for clean and masked responses of the data and that of the simulation. The average deviation is the mean value of the absolute difference between each corresponding discriminability value.

## Results

## Cross-channel lateral inhibition enables the network to match experimentally observed neural responses

As described in Materials and Methods, a multilayer network model (Fig. 2*a*, left) of integrate-and-fire neurons was constructed to replicate selective spatial responses to competing sound sources. Input layer neurons represent neurons at the spatial cue detection level, and receive input generated by the model in Figure 3 when a stimulus is presented at the corresponding location (see Materials and Methods). Thus, there are four input “channels” corresponding to each speaker location in the experiment. The four input units excite four corresponding channels of relay neurons and interneurons in the middle-layer, which inherit their spatial tuning. Relay neurons converge to excite the cortical neuron (Fig. 2*a*, left), making it broadly tuned to stimuli from all directions in the clean (i.e., no masker) case (Fig. 2*a*, inset, discriminability grid), as observed in the data (Fig. 1*a*; see Materials and Methods, Network Model Architecture). However, in this network (Fig. 2*a*, left), the spatial discriminability grid is relatively uniform (Fig. 2*a*, center column), unlike that observed in the data (Fig. 1*d*). Thus, this basic network replicates the broad response in the target alone case, but fails to produce the configuration-dependent hotspots observed in the data.

Introducing lateral inhibition from inter-neurons across spatial channels allows the target response to suppress the masker response when presented at the tuned locations, generating a hotspot of performance for a given target and masker location combination (Fig. 2*b*). Figure 2*d* depicts the subthreshold conductance and voltage changes in the relay and cortical neurons in the expanded time segment. Whereas neuron R2 spikes predictably in response to increases in EPSC, R1 is unable to spike following its EPSC input due to long-lasting suppression by lateral inhibition as seen in the increase in IPSC (Fig. 2*d*, bottom, magenta trace) from I2. In this case, the voltage response of the cortical neuron resembles that of R2 and the 0° target input (Fig. 2*d*). This is seen in the raster plots for the same stimulus paradigm, which resembles the target alone condition (Fig. 2*b*, bottom right), indicating that the cortical neuron is able to follow the target and largely ignore the masker. Note that when the locations of target and masker are reversed, discriminability decreases due to the masking of target by noise (Fig. 2*b*, center and top right). The preferred spatial location combinations in the recorded unit (Fig. 1*d*) can be modeled by introducing additional lateral inhibitory connections as shown in Figure 2*c*.

By adjusting model parameters, we were able to satisfactorily fit 32 of 33 units recorded in the original study. The model was largely robust in the parameter ranges we tested (see Materials and Methods for details). We used two parameters to assess the closeness of fit between each unit and its model simulation. Average deviation measures the closeness of the discriminability values of the simulation compared with the data in units of discriminability percentage, and was 3.39±0.97% for all simulated units. The correlation coefficient ranging from −1 to 1 measures how closely the pattern of the simulated grid agrees with the experimental grid, and was 0.94±0.04 for the simulated units. The neural unit that did not have an overall satisfactory fit had a spatial grid that was very uniform, where discriminability variations within the grid were small and random. As a result, the simulated fit had a deviation value within the normal range, but a very low cross-correlation coefficient.

It is noteworthy that the model network without lateral inhibition showed a relatively uniform spatial grid (Fig. 2*a*, center column), unlike the experimental data. This network did include adaptation and synaptic depression (see Materials and Methods, Model neurons). Thus, without lateral inhibition, adaptation and synaptic depression are not sufficient to explain the experimentally observed hotspots in the spatial grid.

## Spatial tuning

The sharpness of spatial tuning curves was varied to test whether the model can describe the data with broader spatial input at the midbrain level. In the initial simulations, we assumed no crosstalk between spatial channels, which corresponds approximately to a Gaussian spatial tuning curve of width 2σ (twice the SD σ) <15°, where σ is the SD of the Gaussian function. As the width increased, more and more overlap occurred between channels, as shown in the left column of Figure 4*a*.

For the simulations shown in Figure 4, spatial tuning width 2σ was increased to 40°, 80°, and 120°, respectively, while keeping all other parameters identical. The results of broadened tuning widths are shown in Figure 4*b*,*c*. The goodness of fit, as quantified by deviation and cross-correlation coefficient, diminished as tuning width was broadened. The mean and standard deviation of these two measures calculated from the population of simulated units, is plotted as dotted lines and shaded areas in Figure 4*b* for reference. In the 40° case, both deviation and cross-correlation coefficient remain within the range for the population of simulated units. The spatial tuning grid for 40° seen in Figure 4*c* (bottom), also maintains the general features of the data (Fig. 1*d*) and the original minimum overlap simulation (Fig. 2*c*, center). Therefore, this network model remains robust when spatial tuning width is increased to 40°. Even at a spatial tuning width of 80°, which corresponds to a fairly large overlap, the correlation coefficient remains relatively high at 0.91 and the deviation relatively low at 6.42% (Fig. 4*b*). Thus, the model remains robust for spatially overlapping tuning curves, degrading gracefully at very high overlaps (eg, 120°; Fig. 4*c*, top).

## Extending the model network to potential engineering solutions for segregating spatial sound sources

**Figure 5.**
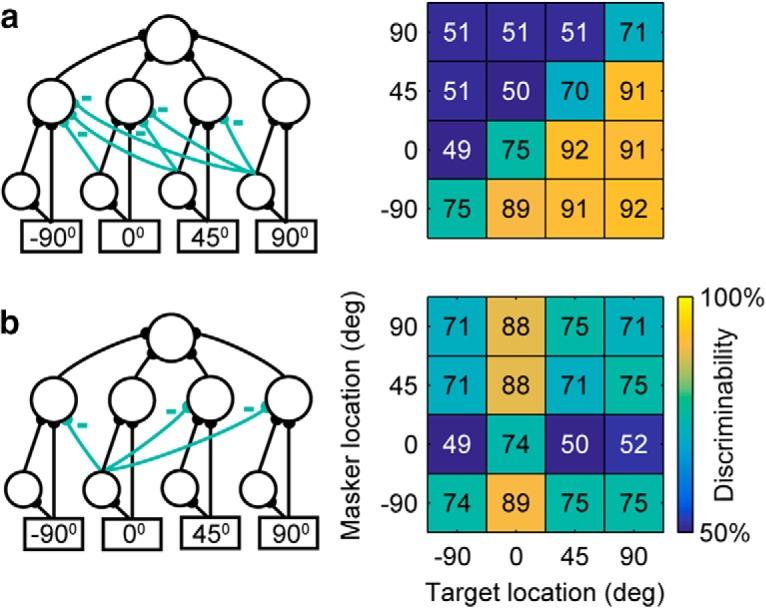
Engineering solutions. ***a***, Left, “Contralateral-dominance” model network where all channels contralateral to the dominant channel are inhibited. Right, Simulation results of this structure achieve the maximum number of spatially separable target and masker locations, where all targets contralateral to masker can be segregated. ***b***, Left, “Beamformer” model network where the channel tuned to the front (0°) inhibits all other channels. Right, The simulated spatial grid illustrating the segregation of the frontal target source.

The network can be extended to provide an engineering solution to the problem of segregating target from noise in space for the maximal number of locations on the grid. Figure 5*a* demonstrates a network where good discriminability is obtained for all conditions with target location to the right of masker location. This network, together with a complementary network with high performance for grid positions above the diagonal, allows the segregation of non-colocated sources for any azimuth, while maintaining consistently high intelligibility when only one source is present. An alternative engineering solution is demonstrated in Figure 5*b*, where one channel acts as a beamformer by inhibiting all other channels. In this case, similar networks beaming at other directions will enable a user to selectively listen to any direction of interest.

## Discussion

The network model used here provides an explicit way of generating neural responses that replicate the key features of the cortical neurons recorded by Maddox et al. (2012), and provides a neural strategy for transforming information into selective coding for sound sources in the presence of multiple sources. The network uses information from input neurons through individual spatial channels and matches the key experimental features through convergent excitation and lateral inhibition across spatial channels.

## Predictions and implications

### Lateral inhibition

The model suggests that lateral inhibition plays an important role in spatial sound source segregation. While lateral inhibition is a widely known mechanism in the brain, to our knowledge this study is the first to demonstrate how it can be exploited in the context of the cocktail party problem. Inhibition is present in field L, as well as the mammalian primary auditory cortex (Müller and Scheich, 1988; Wehr and Zador, 2003). Recently, there has been evidence of suppression by spatially separated stimuli in the cortex of marmoset monkeys (Zhou and Wang, 2012, 2014), which could be a manifestation of the lateral inhibition postulated in the model.

Given this network, we propose a physiological experiment that may provide additional insights. One can experimentally test the nature and source of inhibition by locally blocking GABA receptors and measuring the spatial grid under the same experimental setup. If the recorded spatial grid becomes less spatially sensitive, the proposed lateral inhibitory connections are most likely local.

### Exploring alternate mechanisms for spatial sound source segregation

The above simulations show that the sharpened spatial tuning in the presence of multiple sources, which allows for spatial stream segregation, can be achieved via lateral inhibition across spatial channels. An alternate mechanism for spatial streaming, proposed in a recent study (Middlebrooks and Bremen, 2013) is forward masking. Candidate neural mechanisms underlying forward masking are adaptation and synaptic depression. The network model used here incorporated both of these mechanisms to model the temporal dynamics of the cortical responses. Our simulations indicate that although these mechanisms are important in determining the temporal dynamics of neural responses, they alone fail to produce the diverse spatial grids seen in the Maddox et al. (2012) study because of a lack of cross-channel spatial interactions (Fig. 2*a*, middle). In particular, without lateral inhibition, the model does not replicate the hotspots seen in the experimentally observed spatial grid. Thus, lateral inhibition involving interactions *across* spatial channels is necessary in the model for replicating the spatial properties in the observed data.

### Response to multiple maskers

For each recorded unit, looking at its single-masker spatial grid response provides predictions for how it might respond to multiple maskers. In Figure 2*c*, for example, the simulated neuron is robust to maskers presented from both −90° and 90° (independently) when the target is located at 0°. This is achieved in the model network by inhibitory connections from 0° to −90° and 90°, which means that target stimuli at 0° could mask two simultaneous noise sources from −90° and 90°. Consistent with this intuition, our simulated network for this unit was robust to simultaneous maskers from −90° and 90°. It should be possible to test such predictions by performing two-masker experiments physiologically, and comparing the results to those of single-masker cases for each neuron.

### Potential engineering solution to the cocktail party problem

The engineering solution visualized in Figure 5*b* is robust to simultaneous maskers in all channels other than the target (in this case 3 simultaneous maskers at −90°, 45°, and 90°), making this a particularly attractive design option in the context of hearing assistive devices in the presence of multiple speakers.

We plan to use the proposed engineering solution networks in Figure 5 to segregate mixed-source acoustic stimuli by building a system that can take mixed-source acoustic inputs and output a single desired acoustic source. This will require two additional processing steps. First, a peripheral model that converts acoustic stimuli into neural representations consistent with the network input is needed. This will be a model where neurons selectively respond to a preferred direction using inter-aural cues, similar to previous neural models of spatial tuning (Fischer et al., 2009). Second, the neural network output, ie, spike trains representing the single desired source, needs to be converted back into acoustic waveforms. This can be done using stimulus reconstruction (Mesgarani and Chang, 2012). We are working on both steps with the long-term goal of ultimately testing the segregation capabilities of the model on normal and hearing-impaired listeners.

### Spatial tuning of inputs and applicability of model to spatial processing in birds and mammals

For the majority of simulations, input neurons are assumed to have non-overlapping Gaussian spatial tuning curves centered at azimuths corresponding to those used in the experiment. A separate set of simulations showed that the model network remains robust when the spatial tuning curves are broadened to have significant overlap.

Spatially selective neurons found in the owl midbrain (Knudsen and Konishi, 1978; Peña and Konishi, 2001) and chicken hindbrain (Köppl and Carr, 2008) demonstrate ITD sensitivity within the physiological range. Although spatial tuning of midbrain neurons in the zebra finch remains unknown, it is likely that the auditory periphery contains similarly spatially sensitive neurons like other avian species, as spatial tuning appears to follow an evolutionary divide across species (Schnupp and Carr, 2009; Ashida and Carr, 2011). An outstanding question is whether the model will hold for species whose midbrain neurons show broader spatial sensitivity, such as small-headed mammals where tuning curves span an entire hemisphere or more (Vonderschen and Wagner, 2014). As we tested, the selective mechanism remains robust when spatial tuning is widened up to 40° (Fig. 4), comparable with some azimuth ITD tuning functions recorded in the rabbit IC by Day et al., (2012).

In species that show broad spatial tuning in the midbrain, spatial tuning may be further sharpened within the cortical level. One possibility is that broad spatially tuned precortical inputs are sharpened by a high threshold at the cortical level. A second possibility is that the spatial tuning of cortical neurons is sharpened during active engagement in a task (Lee and Middlebrooks, 2011). In this case, the authors proposed a top-down activation of inhibitory mechanisms as a potential mechanism. The Maddox et al. (2012) experiments were in an anesthetized preparation, so lacking top-down activation, but it is possible that sharpening of tuning via lateral inhibition can be elicited by top-down activation (eg, during active engagement), or bottom-up activation (eg, in the multiple source condition). A third possibility is that for neurons with broad spatial tuning, the hypothesized spatially tuned inputs may be achieved through population coding, ie, computations based on effective pooling across input neurons.

The neurons in the experiments by Maddox et al. (2012) were recorded in field L of the zebra finch, the analog of mammalian primary auditory cortex. Although the strict homology between auditory areas in birds and mammals is still debated, the functional properties of Field L neurons, eg, spectrotemporal receptive fields, are similar to those observed in mammalian auditory cortex (Sen et al., 2001). In addition, the trend of less spatial specificity for single sources from primary spatial cue detection areas to higher cortical areas appears common across mammalian and bird species (Vonderschen and Wagner, 2014), for which this study provides a possible explanation. Thus, the model described here may explain some of the general properties of cortical neurons in other systems.

### Population coding and readout

The network presented here suggests that in the presence of multiple sound sources, cortical neurons can “selectively listen” to particular target sources, which correspond to hotspots of performance on the spatial grid. A population of such neurons, for different locations in space, would enable spatial streaming over a range of locations. This is consistent with the diversity of spatial grids with hot spots at different locations observed in the experimental data (Maddox et al., 2012). The experimental data were obtained in anesthetized animals, suggesting that such a population representation is “pre-attentive”. Attention may facilitate the proper readout from this cortical population by selecting the appropriate neuron(s) for given target and masker locations.

### Concluding remarks

In this study, we presented a computational model describing how the auditory cortex may transform spatial representations to solve a key aspect of the cocktail party problem. The computational model is based on physiological data (Maddox et al., 2012) and makes two key predictions that can be tested experimentally. First, the model predicts that lateral inhibition is a core mechanism underlying spatial sound source segregation. It would be interesting to further elucidate the nature and the location of such inhibition in similar experiments by pharmacologically blocking local GABA receptors. Second, the model predicts that some cortical neurons will remain robust when additional maskers are added in select locations predicted by the model. This can be tested in experiments on spatial selectivity of cortical neurons with three or more sound sources.

In addition to testing these key experimental predictions, it will also be interesting to implement the engineering solutions discussed in the paper and test whether the proposed circuit can successfully segregate sounds sources and improve listening performance in normal and hearing impaired listeners in cocktail-party-like settings.

## Synthesis

The decision was a result of the Reviewing Editor Tatyana Sharpee and the peer reviewers coming together and discussing their recommendations until a consensus was reached. A fact-based synthesis statement explaining their decision and outlining what is needed to prepare a revision is listed below. The following reviewers agreed to reveal their identity: Jose Pena, Heather Read

In the revised manuscript please pay particular attention to the following two issues. First, please justify the model set up that you use, such as the use of receptive fields that do not overlap in space and the inclusion of inhibitory neurons in the thalamus. It might be possible to justify these receptive fields as representing effective outputs of computations at the population level even if none of the actual receptive fields in the population does not have these properties. Second, please comment on the model robustness to the heterogeneity in properties of spectrotemporal receptive fields. Please also note that a visual abstract is required. Reviewer comments:

Reviewer 1:

This paper proposes a model to explain the space-dependent song selectivity in the presence of concurrent sounds, published in Maddox et al. 2012. The model is based on lateral inhibition across relay neurons tuned to non-overlapping directions. Adding lateral inhibition makes it possible to predict how neurons in the avian auditory cortex receiving convergent projections from the relay neurons, may respond to an individual song more precisely when it comes from a particular direction, in the presence of masking sound.

The paper is nicely written and provides a theoretical solution to an interesting phenomenon, suggesting ways to test it. On the other hand, it is largely hypothetical and most assumptions are not substantiated by data. The paper explains a phenomenon, provided these assumptions are true. The paper parsimonious about conclusions and does not hide these caveats. As is, it may constitute a nice first step into a working hypothesis.

My general and specific comments are listed below.

General comments

The critical assumption is the non-overlapping spatial tuning of convergent inputs. As I understand, each input cell would be broadly tuned to frequency and fairly sharply tuned to space. Whether such inputs to the auditory cortex exist in the avian or mammalian brain, is not known. The necessity and sufficiency of lateral inhibition to explain Maddox et al 2012 data occurs only if these assumptions are true. The paper is frank and straightforward about it, but it nevertheless sets a narrower scope than if it demonstrated the necessity of lateral inhibition unequivocally.

The alternative hypotheses are only addressed in the discussion. The control test, without lateral inhibition, implicitly tests some alternative hypotheses for generating the selectivity, such as adaptation and synaptic depression. I think being more explicit about this in the results, could make the paper stronger.

The abstract and discussion mention interaural time different (ITD) as a possible source of spatial tuning. ITD selectivity is determined by the ability of neurons to phase lock, which only exists at lower frequencies. Thus ITD-driven spatial selectivity will be restricted to low frequency sounds. On the other hand, the best frequency reported in Table 2 is 4300Hz. This frequency is likely above or in the upper range of ITD selectivity of zebra finches. If based on ITD, this high best frequency will act against the narrow spatial tuning.

I found figures and figure legends a serious impediment for grasping the paper. Figures are not self explanatory and legends are not descriptive enough of the figures.

Specific comments

1. As I understand from the methods and Table 2, STRFs' parameters were rather fixed, in terms of bandwidth, best frequency, etc., across input neurons. The STRF parameters were based on Amin et al 2010, which compares midbrain, thalamic and Field L neurons in the zebra finch. I understand that paper showed heterogeneity in spectrotemporal tuning in both the thalamus and Field L. How robust is the effect of lateral inhibition to changes in STRF parameters?

2. Table 1 legend uses the STRF abbreviation and is called before STRF is defined in the text. Then the STRF abbreviation is defined twice, once in the text and once in a subtitle.

3. Figure 5 is called before Figure 4 in the text.

4. The tables are not described in the legends. Some of the parameter abbreviations used in table 2 appear different from those defined in the text.

5. Figure 1. Panel b is labeled as d. The description of what each panel shows is insufficient. The row with discriminability indices is defined in b, but it also applies to a and c. What are the M's with arrows in panel b (d)?

6. The terminology 'multiple lateral inhibitions' is slightly confusing. I suggest 'lateral inhibition from multiple...'

7. Figure 2: Panels a and b are overlapping, which makes it difficult to follow the legend. The legend should say what the solid and dashed squares are, in the indices grids.

8. Figure 3's legend is just one uninformative sentence.

9. Figure 4's legend makes conclusion statements, rather than describing panels a and b.

10. Figure 5: I think it is better to label the panels on the top of the figure. The legend is not descriptive enough. It is hard to go from what the legend says about panel b to what the figure shows. For panel c, there are statements about similarity of patterns, as if it were obvious to readers.

Reviewer 2

This study explores plausible/feasible properties that allow networks and individual neurons to discriminate sounds in noisy acoustic scenes and could have far-reaching theoretical implications and engineering applications. Unfortunately, in its current form it will be lost on most readers of eNeuro as it covers a lot of territory (many simulation variations). I think this could be remedied by some careful revision.

Line 40, text, "Spatial tuning of neurons for single sound sources in higher brain regions such as the cortex is broader and not topographically organized Stecker et al., 2005" I am not even sure that this statement needs to be there given your simulations but if you want to say this you should not cite a Stecker et al. alone. There is some debate as to whether spatial tuning is topographically organized in auditory forebrain and likely there are meaningful species differences. Many papers have addressed this issue using full or partial virtual acoustic space cues. For example Campbell RA, Schnupp JW, Shial A, King AJ. J Neurophysiol. 2006 found a distributed non-topographic organization within a single auditory field of ferret. Conversely, Semple MN, Kitzes LM. J Neurophysiol. 1993 find response clustering and topographic organization of binaural intensity tuning within primary (A1) auditory cortex of the cat. Higgins et al., J Neuroscience 2010 find an organization of binaural intensity tuning that shifts systematically from highly lateralized (more contralateral) to more midline as you move from one cortical field to the next -which is a form of topography not unlike that described by Semple and related papers on the cat A1.

Line 61, no need to refer readers to Methods in the Introduction.

Line 60, "A larger difference in neural responses to the two songs gives higher discriminability, indicating a location where birdsong is more "intelligible" to the neuron." Do you mean spatial or song discriminability or a joint spatial-spectral-temporal discriminability that could be used to discriminate songs. Phrasing is not clear here.

Line 69, In the Introduction here you use the phrase "sharpened spatial preference" which is OK and consistent with the notion that responses shift from almost uniform 100% rate across all spatial positions (Fig. 1d, clean) to a more limited range of positions with response maximum when the masker is present (Fig. 1d, masked). However later, in line 76 and line 215, you talk about how the "network can be tuned to model.."

and say, "By tuning model parameters (see Methods), we were..." Could you not use the word tuning here please? It is less confusing if you reserve that term to something well defined such as the Gaussian model of your azimuth response tuning width/shape or spectrotemporal receptive field (STRF) tuning bandwidth.

Methods, Line 90, you jump into describing the network architecture which primarily involves interneurons and relay neurons in thalamus and their convergence onto cortex and yet you don't really give a rationale. Please quote physiologic data supporting the rationale for the architecture, the particular short term depression values, the thalamocortical relay neuron convergence factors. I see several aspects that could be clarified. First, your simulated network includes a variable for short-term suppression plasticity and you quote Varela et al, a computational model of visual pathways. Short-term depression is also a real factor in auditory thalamocortical networks (e.g. Reyes coauthors and others) and this is a great rationale for implementing it in your model even if that wasn't your actual starting point. Second, I see no rationale for modeling inhibitory interneurons in the lemniscal thalamus of the bird. Please quote the appropriate literature. Birds do have something akin to a reticular nucleus with inhibitory neurons but people generally think of this inhibition as being driven by cortico-thalamic pathways that would have very different convergence and connection patterns than the architecture used here. It is OK if you implement a hypothetical thalamic interneuron as it worked for you and in truth some mammals have these (see Winer and colleagues for example show rodents don't have lemniscal inhibitory neurons but cats do). A third area needing clarification is why you model spatial response fields or "source azimuth with essentially no overlap.." (see below line 197). Having recorded and modeled many azimuth tuned responses I find this to be biologically implausible though it is clear from this study that it works for your purposes so please explain its feasibility and caveats. Though you do explain that you simulate spectrotemporal receptive fields (STRFs) with parameters obtained in the zebra finch by Amin et al., 2010). You do not explain your rationale for azimuth tuning parameters or quote appropriate literature (Gold and Knudsen, owls, Higgins et al, rats, Stecker Middlebrooks, cats Semple for primates) . You should more clearly describe your network parameter starting point ranges and rationale for use. Remember, it is fine if your simulated network is really more like a cat than a bird because your network nicely recapitulates the "hot spot" phenomenon observed in real auditory neurons when noise maskers are present. These clarifications in the Methods will make it easier for other scientists to make sense of this model and will make it seem less idiosyncratic.

Line 149, Table 2. In this table you include one simulation with a bandwidth of 2000 Hz. So are you saying that all the STRFs in the simulated network had a fixed bandwidth of 2000 Hz? Also, it is not clear is whether when using the STRF bandwidth with "minimally overlapped azimuth tuning" whether you also are using minimally overlapped spectral tuning? In real systems (including songbirds as you know) there is some degree of spectral overlap between neighboring neurons with STRFs moreover since the STRF consists of temporally precise (phase-locked) responses correlations exists between neurons with spectral centers that are shifted by >= about 1/3 an octave (e.g. Chen et al. in the inferior colliculus). It is not clear whether this contributes to the effects you see.

Methods, Line 125-127, "For the majority of simulations spatial tuning was assumed to be represented by Gaussian functions of source azimuth with essentially no overlap.." Do you mean to say something more like, "the azimuth response field for each modeled neuron was simulated with a Gaussian function and across the population there was minimal overlap between response fields."

Line 160, "Spatial tuning width at the midbrain level varies across species, and is notably broader relative to the physiological range for some mammals Vonderschen and Wagner, 2014)." Not sure what this statement means. Do you mean the neuronal spatial tuning is broader than the behavioral tuning?

Line 161, "To investigate whether the network model is functionally feasible in animals with broader spatial tuning, the effect of spatial tuning width was studied...." An animal has spatial discrimination or detection differences not "spatial tuning".

Line 191, "These input units send excitatory synapses to relay neurons and inter-neurons, which inherit their spatial preferences.." In the Methods, please explain a bit more clearly what you are modeling and cite that here.

Line 192-193, "Relay neurons convergently excite the cortical neuron (Fig. 2a, left).." I love this word "convergently" but I am not sure it is a word. More importantly, please explain how you determine your network convergence architecture in the Methods and cite appropriate literature and refer to that part of Methods in this section of Results.

Discussion, Line 334, assuming you are referring to interaural level difference cues (and not timing cues) for azimuth you should quote the most appropriate of Kuwada et al., J Neurophys 2011, Front Neural Circuits, 2012 here in addition to Day whom you already quote.

Discussion, paragraph beginning with Line 279, this is an excellent paragraph. I would like to see you elaborate on this a bit.

General Discussion. Since I found it hard to follow your logic or rationale for your simulated network architecture parameters (convergence factors, inhibitory interneurons, non-overlapping azimuth and STRF responses) during the body of the paper, it is hard to weed through this Discussion. Once you clarify all of that in your Methods, then it would be nice to more clearly walk the reader through the significance of the lateral inhibition as you begin to do on Line 279.
